# On Contour

**Published:** 1895-05

**Authors:** T. G. Huntington

**Affiliations:** New York


### ON CONTOUR.[1]

BY DR. T. G. HUNTINGTON, NEW YORK.

Mr. President and Members,—Much has been said and written in regard to the use, durability, and comparative merits and demerits of each and all filling-materials which are and have been in use for the purpose of arresting caries. So thoroughly has this subject been discussed that we sometimes feel that the last flaw has been found and the last virtue recorded, and that an end of the whole matter has been reached. Then some one puts forward a new form of an old material, or an old material with a new name. We try it, and commend or condemn it according as it stands our test.

Whether the material is new or old, commended or condemned, the discussion which takes place is fruitful and with some good results.

While filling-materials are still made the subject of discussion, it may not be amiss to review that which has to do with the efficiency of all filling-materials,—*i.e.*, form or "contour."

What I say upon this subject may for the most part apply to

---

[1] Read at a meeting of the New York Odontological Society, November 20, 1894.

all teeth, but I have in mind more particularly bicuspids and molars. The surfaces of all fillings are either flat or convex, and to make a flat filling is comparatively easy; but to restore the lost contour of a tooth with any of the filling-materials is quite another thing, and from beginning to end requires forethought and care, a greater expenditure of time, labor, and material. Therefore, if a dentist is to spend the best part of his life making fillings, why should he make convex or "contour" instead of flat fillings.

Whatever the agency which acts upon a tooth to first produce caries, that agency is no less potent, it is reasonable to suppose, after the carious cavity has been filled than it was before. If accumulation of decomposing foods has been the cause, a flat filling of any size affords a better lodgement for such deposits, and the efforts to cleanse the tooth will be less effectual after such a filling than they were when the convex surface of the tooth was presented; especially is this so when, as is usually the case, a V-shaped space is left, the angle being towards the neck of the tooth.

To keep such a space cleansed requires more vigilance than any one is likely to exercise, and the result is sure to be that the filling gives way, at the same time involving the surface of the approximal tooth. Indeed it is probable that this takes place before the first filling shows any signs of failure, and the point of decay comes so near the cervix that to fill the second cavity requires a great deal of cutting away of the convex approximal surface. When the work is done the teeth or fillings, or both, will be in contact from grinding surface to gum, or there will be a V-shaped space, which is worse.

Every dentist knows what then takes place, so far as these two fillings are concerned. The case would not be so bad, however, if the only evil resulting from flat fillings were to limit the period of their own usefulness.

The V-shaped space readily admits all sorts of material, fibrous and otherwise, and holds it. More presses upon it until the gum is forced away from its natural position. Particles of food become lodged under the gum, causing irritation and suppuration. It is by no means uncommon to see a cavity thus formed extending half way from the neck of a tooth or teeth to the apex of the roots. The alveolus and peridental membrane give way, and if there is any tendency to calcareous deposits, they find their way most readily to this secluded place and then carry on the destructive work. I believe that a great deal of the trouble which comes to the surrounding tissues of the necks and roots of teeth owes its inception to the very process which I have described. I have chosen approximal surfaces of bicuspids and molars because I have always considered that these teeth, and especially the bicuspids, are difficult to fill, and fill correctly and permanently as any in the dental arch.

When a material or a method of manipulation makes secure against the inroads of decay the gum margins of approximal bicuspid fillings, that material or manipulation will be successful anywhere in the mouth.

There are in each arch of the adult mouth eighteen such surfaces, which are particularly liable sooner or later to require filling. We all frequently see cases where at least half this number of surfaces are either filled or require filling. Now, if all these teeth by reason of their filling lose something of their width, the aggregate loss in each arch would, at the very least, amount to the width of one molar. The contraction which results would perhaps be as great as though a first molar were extracted from each side. It is unnecessary to more than hint at the result of such an operation. The contracting arch first changes the direction of the crowns of the teeth as well as their normal position. This change disarranges the articulation of the entire set. This disarrangement through contraction, whether it occur by the loss of teeth or from the cutting away incident to frequent renewing of filling, produces an extreme and unnatural wearing of the grinding surface of the molars. The wearing shortens the bite and prevents the free motions of mastication. It also occasions a moving forward of the lower arch, which brings undue wear on the cutting-edges of the incisors, and also brings a force upon the bicuspids in mastication which when they are weakened by large fillings they are unable and never intended to support.

It may be questioned whether a flat filling need to detract from the original width of any tooth. I would simply say that it is not necessary, but is it not a fact, take them as we find them, that they do?

At this point it would be very easy to touch upon the occasion of flat fillings and why it is that they continue to be made. Undoubtedly a good many fillings which are started as contour fillings turn out to be flat ones. The contour being somehow lost in the process of finishing.

Some things must be defined negatively; and one way of emphasizing the necessity of the use of *contour* filling is to indicate some of the evils resulting from the lack of it.

The first requisite of contour filling is plenty of space in which to work. To get this requires a little time,—perhaps a week. Sometimes, however, when a tooth has lost its normal position by reason of a large approximal cavity in the tooth next to it, the wedging process is more of an operation.

How much cutting away should be done in the preparation of cavities is a question which probably might give rise to a great deal of variety of opinion and practice. My own theories and practice have been most in accord with those who believe in pretty free cutting of all frail portions, or portions which, unless cut away, will, when the cavity is filled, bring the line between filling and enamel in contact when the filling is finished with the neighboring tooth. The sacrifice of enamel and dentine sometimes seems unnecessary, but thus far in my own experience I have failed more often from the lack of free cutting than I have from the contrary.

If there were more cutting away of the lateral margins of approximal cavities at the cervix of teeth, I firmly believe that we would have less fault to find with the efficiency of filling-materials.

Where amalgam is used, is it not a fact that frailer walls are allowed to stand at the cervix than in the use of tin or gold ; and does this not amount, in part, for its seeming lack in efficiency ?

I have used the terms "convex" and "contour" in somewhat close association. They are not, however, synonymous. A contour filling neither adds to nor detracts from the normal size or shape of a tooth. When the arch is unbroken as far as the teeth may extend from the median line back, my plea is for "contour" filling.

The word itself and our knowledge of the relationship of each tooth with the others of the arch and both arches fixes the point of contact of approximal as well as antagonizing teeth.

In conclusion I would ask, How do you fill a pin-head cavity in the approximal surface of a molar or bicuspid ?

